# Quantitative prediction of the effect of genetic variation using hidden Markov models

**DOI:** 10.1186/1471-2105-15-5

**Published:** 2014-01-09

**Authors:** Mingming Liu, Layne T Watson, Liqing Zhang

**Affiliations:** 1 Department of Computer Science, Virginia Polytechnic Institute and State University, Blacksburg, VA, USA; 2 Department of Mathematics, Virginia Polytechnic Institute and State University, Blacksburg, VA, USA

## Abstract

**Background:**

With the development of sequencing technologies, more and more sequence variants are available for investigation. Different classes of variants in the human genome have been identified, including single nucleotide substitutions, insertion and deletion, and large structural variations such as duplications and deletions. Insertion and deletion (indel) variants comprise a major proportion of human genetic variation. However, little is known about their effects on humans. The absence of understanding is largely due to the lack of both biological data and computational resources.

**Results:**

This paper presents a new indel functional prediction method HMMvar based on HMM profiles, which capture the conservation information in sequences. The results demonstrate that a scoring strategy based on HMM profiles can achieve good performance in identifying deleterious or neutral variants for different data sets, and can predict the protein functional effects of both single and multiple mutations.

**Conclusions:**

This paper proposed a quantitative prediction method, HMMvar, to predict the effect of genetic variation using hidden Markov models. The HMM based pipeline program implementing the method HMMvar is freely available at https://bioinformatics.cs.vt.edu/zhanglab/hmm.

## Background

Genomic variability contributes to evolution and population diversity. With the development of high throughput technologies, a massive amount of variation data is available in online public databases, for example, dbSNP [1], dbVar [2], Human Gene Mutation Database [3], Ensembl [4], and Catalogue of Somatic Mutations in Cancer (COSMIC) [5]. Different types of variation have been identified, such as single nucleotide polymorphisms (SNP), short sequence repeat, insertion/deletion polymorphism (indel), copy number variants (CNV), and inversions. Recent pilot studies from the 1000 Genomes Project Consortium [6] and the International HapMap Project [7] revealed that there are about 15 million SNPs, one million short indels, and 20,000 structural variants (SVs) harbored by the studied populations.

Indels, especially frame shifting insertions and deletions, are expected to have large effects on protein functions, since they may change the reading frame of a gene thus change amino acids and probably the functions of proteins. It has been shown that indels cause more severe functional changes in proteins than SNPs [8] and also have significant influence on protein-protein interaction interfaces [9]. As revealed by the Human Gene Mutation Database [3], approximately half (57%) of the human (gene sequence level) disease variations are associated with single nucleotide substitutions, and about a quarter (22%) are associated with small indels [3,10]. Mill et al. [11] have shown that 42% of the nearly two million indels they identified are mapped to human genes and more than 2,000 indels affect coding exons and likely disrupt protein function and cause phenotypic change in humans. Moreover, they found that many of the identified indels had a high level of linkage disequilibrium (LD) with SNPs, which indicates the indels might be the essential factors that cause diseases. Furthermore, indel variants have profound functional impact in human specific evolution and adaptation [12-14].

With an increasing amount of genomic variability data, computational tools for prediction of the functional impacts of these variants on proteins are needed to help biologists select variants for experimental studies. So far, SNPs have been intensively studied and tools for predicting SNP functional effects have been developed, while little is known about the functional effects of indels, the second most common type of genetic variation in humans.

The protein sequence based prediction methods for functional effects of different types of variants are typically grouped into two classes [15], constraint based predictor and trained classifier. Previous studies mainly concern SNPs and there are a few dozen computer programs and web servers devoted to predicting the effects of SNP variants. For example, SIFT SNP [16] is a constraint based predictor and PolyPhen [17] is a trained classifier, both protein sequence based. There are also many nucleotide sequence based prediction methods using evolutional information, such as GERP [18], SCONE [19], etc. In contrast, the efforts devoted to indel effect prediction are limited. Recent indel prediction studies include an evolutionary conservation based approach for both coding and noncoding regions [20], a trained classifier method for frameshift variants [21], and another evolutionary conservation based method for multiple types of variation [22]. This paper proposes a profile hidden Markov model (HMM) [23] based approach HMMvar, which differs from previous approaches in having a formal probabilistic basis.

**Figure 1:**
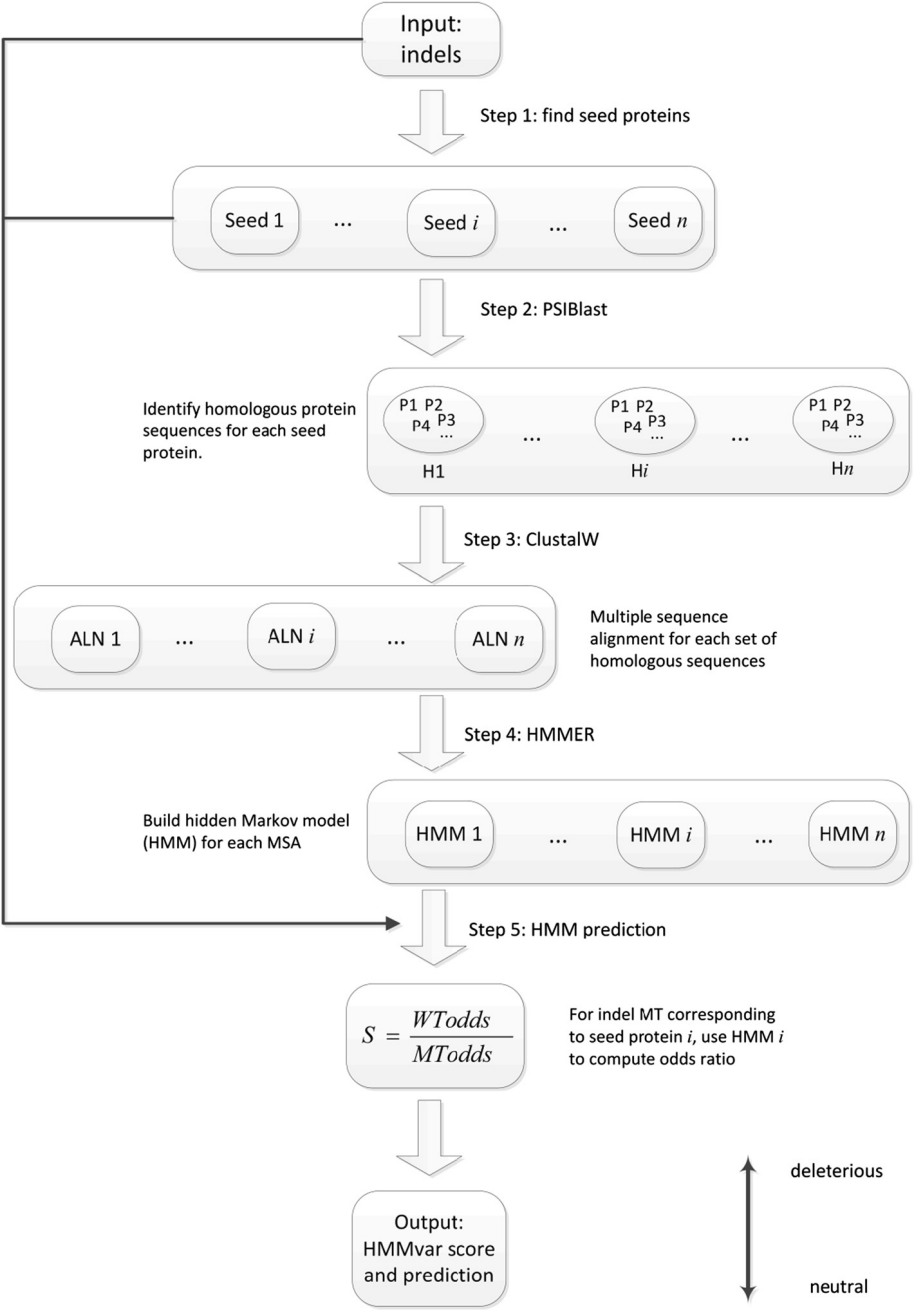
A pipeline of variant prediction using HMMvar.

A *profile HMM,* named for the characteristic output "profile" of a particular hidden Markov model (HMM), is a finite state machine consisting of a series of nodes, each of which corresponds roughly to a position (column) in the alignment from which it was built. Most of the previous prediction methods are based on the principle that important amino acids will be conserved in the protein family, and so mutations occurring at well-conserved positions tend to be deleterious to the functions of the protein. This is exactly the feature of profile HMMs. Basically, a profile HMM is a probabilistic description of the consensus of a multiple sequence alignment. Thus it is reasonable to consider profile HMMs as a tool for predicting functional effects of variants. A flowchart of profile HMM based prediction is shown in Figure 1. The pipeline basically consists of five steps: 1) find "seed" proteins that are associated with indels; 2) for each seed protein, find homologous sequences from a database; 3) do multiple sequence alignment (MSA) for each set of homologous sequences; 4) build a profile HMM based on each MSA; 5) predict the functional effects of indels using the profile HMMs (see Methods for details).

## Results

### HMMvar prediction of indels

Indels were obtained from the database dbSNP Short Genetic Variants [1], including human coding nonsynonymous mutations, such as nonsense, missense, and frameshift indels. *Nonsense* means the mutation introduces a stop codon, for example, the codon TCA changes to TGA. *Missense* means the indels that add or remove amino acids to or from the original protein sequence, for example, the codon ACT changes to GCT, which alters threonine (Thr) to alanine (Ala). The length of a missense indel is always divisible by three, which means the sequence is still in frame with the variants. A missense SNP is an SNP that leads to the replacement of the original amino acid with a new one. *Frameshift* means the mutation changes the open reading frame of protein translation. The data is then classified into two groups: variants that have Locus-specific Mutation Database (LSDB) [24] annotation, which are expected to be disease associated and have more harmful effects, and variants that do not have LSDB annotation, which are expected to be nondisease (or unknown) associated and have less harmful effects. Since the amount of LSDB indel and nonLSDB indel in the database is highly imbalanced, we randomly sampled the same number of proteins that have indel mutations in both categories. Table 1 lists the indel categories of the dataset. The fractions (4% and 95.7%) of nonsense and frameshift mutations in the LSDB group are higher than those (1% and 95.1%) in the nonLSDB group, while there are no missense indels in the LSDB group but 56 in the nonLSDB group, suggesting that nonsense and frameshift indels are more likely to cause diseases.

**Table 1:** Dataset from dbSNP

	**LSDB**	**NonLSDB**	**Total**
Nonsense	112	15	127
Missense	0	56	56
Frameshift	2519	1387	3906
Total	2631	1458	4089

**Figure 2:**
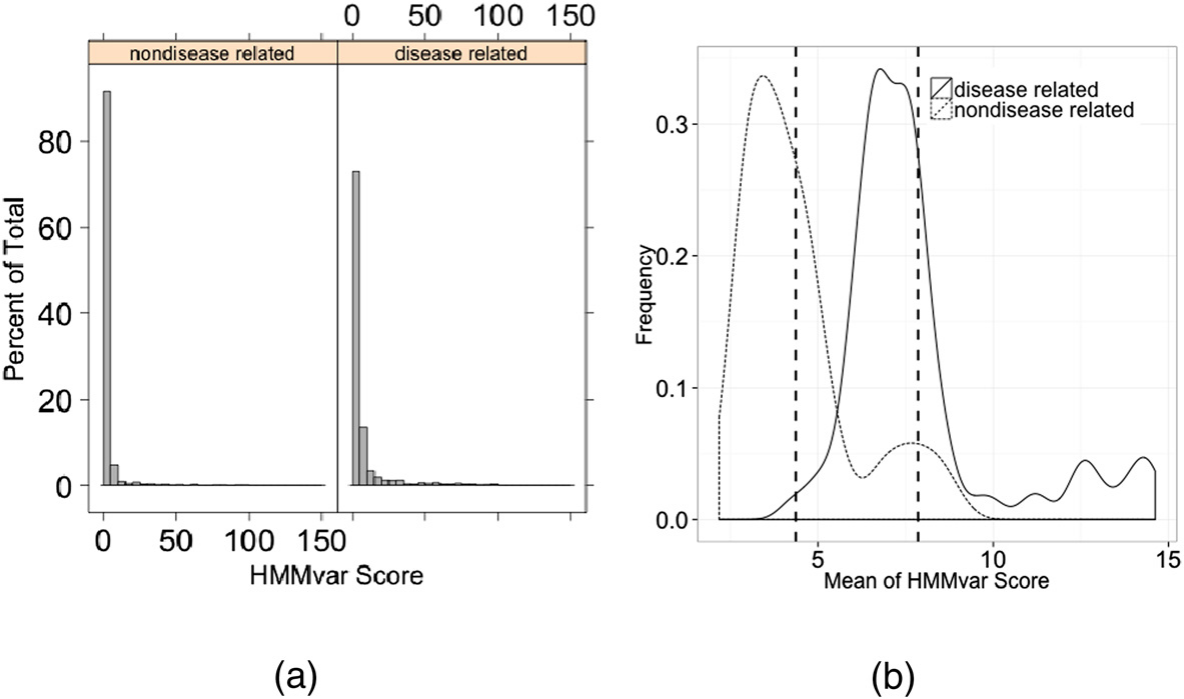
**HMMvar score distribution of the dbSNP dataset. (a)** Histogram of HMMvar scores for disease associated indels and nondisease associated indels. **(b)** Distribution of sample means of HMMvar scores from the two categories (LSDB and nonLSDB).

The effects of indels in these two groups (LSDB and nonLSDB) were quantified by HMMvar. Figure 2(a) shows the distributions of the HMMvar scores (the odds ratio, S, described in the Methods section) in the disease associated and nondisease associated groups. When the score is small (typically *S* < 1.4), nondisease associated variants dominate, while disease associated variants significantly dominate the right side of the distributions (S ≥ 1.4). There is a significant difference between the HMMvar score distributions of the two groups (Kolmogorov-Smirnov test, *p* < 2.2e–16). The mean scores in the two groups were compared by a one sided two sample t-test where 200 variants from each group were randomly sampled with replacement and the means of the sampled data from the two groups were compared. This process was repeated 100 times, yielding two distributions of the sample means as shown in Figure 2(b). The two vertical dashed lines represent the means of these two distributions, which are significantly different (t test, *p* < 2.2e–16).

**Figure 3:**
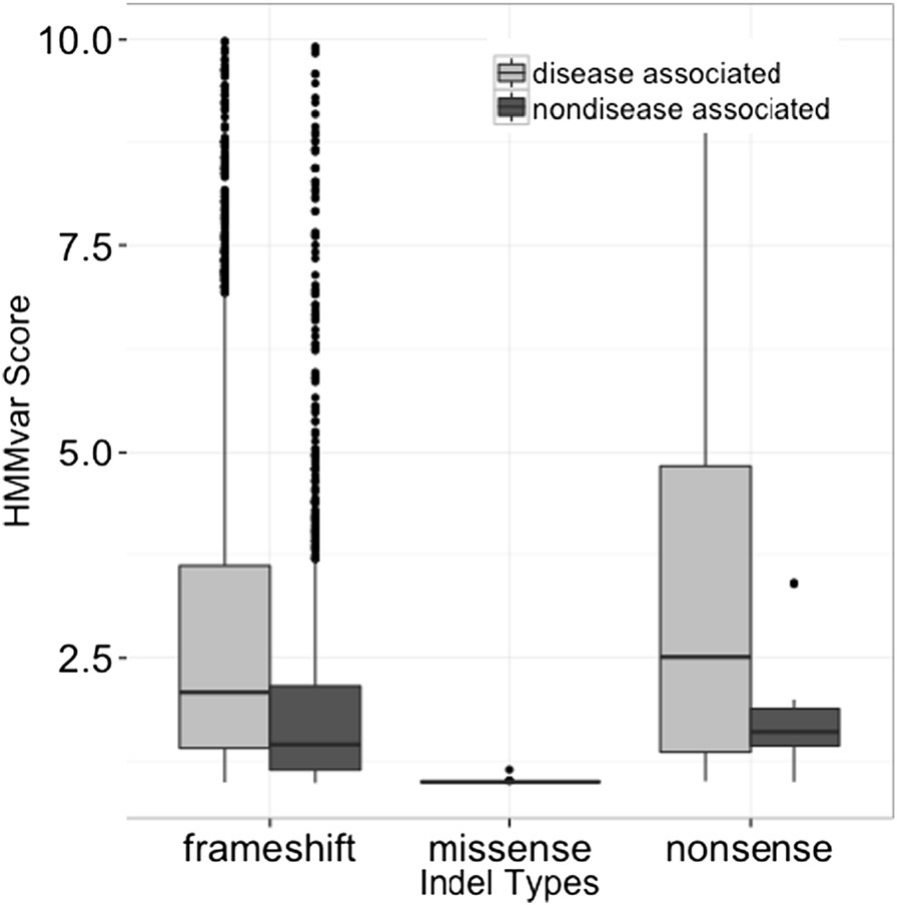
Distributions of HMMvar scores for different types of variants.

Different functional types of variants (nonsense, missense, and frameshift) were combined to give an overview of the distributions of the HMMvar scores for different groups (Figure 3). The most remarkable feature is that the score of missense indels is much lower than the scores of the other two types, consistent with the notion that missense mutations tend to have less deleterious effect than frameshift indels and nonsense mutations. In each type of indel, the median of the nondisease associated group is lower than the median of the disease associated group, demonstrating that the HMMvar score is effective in measuring the deleteriousness of indel mutations.

To test the consistency of HMMvar scores with a genome wide analysis, the indels with minor allele frequency (MAF) in dbSNP were extracted, resulting in 447 indels to be scored. The less the allele frequency is in a certain position of a genome, the more conserved the site and the more deleterious the effect of a mutation at this site, in terms of evolutionary theory. In this experiment, the MAF shows a negative Pearson correlation with the HMMvar score (r = –0.03), which is consistent with the common indication of MAF (the lower the MAF, the higher the significance of the site), though the correlation is not significant.

### Comparison with other tools

**Figure 4:**
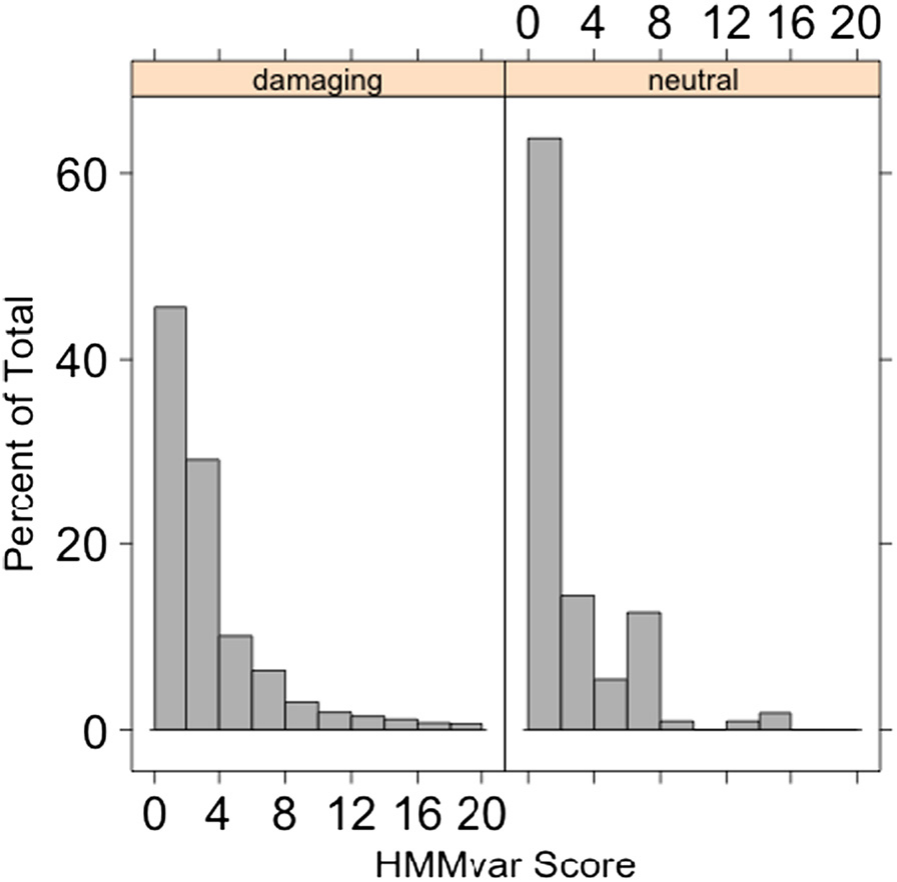
**Compare HMMvar prediction with SIFT Indel prediction on dbSNP indel dataset.** Distributions of HMMvar of indels that are predicted as damaging (left) and neutral (right) by SIFT Indel.

This section compares HMMvar with SIFT Indel [21], a tool recently proposed for predicting indel effects, and two commonly used effect prediction tools for SNPs only, SIFT SNP [16] and PolyPhen [17]. SIFT Indel uses a trained classifier (decision tree) method to predict the effect of indels. Four features were extracted for each indel: 1) fraction of affected conserved DNA bases; 2) indel location relative to a transcript, taking the maximum across all transcripts; 3) fraction of affected conserved amino acids, taking the maximum across all transcripts; and 4) minimum distance of indel to the exon boundary of all affected transcripts. The classifier was then trained based on the training data. Though easy to interpret due to the nature of a decision tree, the predictive power is limited because the classifier only applies to frameshift indels, which account for a tiny proportion (~ 0.05%) of all indels, and it provides only a coarse grained qualitative prediction, either "damaging" or "neutral", rather than a quantitative measurement. Figure 4 shows the distributions of HMMvar scores of two groups, "damaging" and "neutral", predicted by SIFT Indel on all the frameshift indels shown in Table 1. They have significantly different distributions (Kolmogorov-Smirnov test, *p* = 2.273*e*–09), indicating that the HMMvar score is able to predict the two different functional effects using SIFT Indel prediction as a reference. When the score is small (typically *S* < 2), the frequency of neutral indels is higher than the frequency of damaging indels. On the other hand, when the score is large *S* ≥ 2, the frequency of damaging indels dominates. Three Fisher’s exact tests were done: 1) HMMvar prediction vs. SIFT Indel prediction, 2) HMMvar prediction vs. database annotation, and 3) SIFT Indel prediction vs. database annotation. The p-values are 7.778e-05, 3.456e-12, and 0.4863, respectively, showing that HMMvar prediction has higher correlation with database annotation. The sensitivity, specificity, and accuracy comparisons between HMMvar and SIFT indel are shown in Table 2. SIFT Indel prediction has higher sensitivity but very much lower specificity than HMMvar prediction.

**Table 2:** Comparison between HMMvar prediction and SIFT Indel prediction with dbSNP indel dataset

	**Sensitivity**	**Specificity**	**Accuracy**
HMMvar	77.8%	68.6%	77.7%
SIFT Indel	95.7%	5.9%	94.0%

Both SIFT SNP and PolyPhen are prediction tools for nonsynonymous SNPs only. To compare with these two programs, SNPs were downloaded from the database ENSEMBL (version: Variation 69, GRCh37.p8), along with precomputed scores and predictions. Among the more than one million SNPs downloaded, only about 80,000 SNPs have Polyphen and/or SIFT predictions. There are two SIFT SNP prediction categories, deleterious and tolerated, and three PolyPhen prediction categories, benign, possibly damaging, and probably damaging. Since prediction for SNPs is very time consuming due to the PSIBlast database searching, 393 SNPs were randomly selected as shown in Table 3. To balance the data, PolyPhen’s possibly damaging and benign categories are combined together. Fisher’s exact test for the HMMvar prediction (cutoff 1.002) vs. the SIFT SNP prediction has p-value 5.626e-05, HMMvar prediction vs. PolyPhen prediction has p-value 0.2285, and SIFT SNP prediction vs. PolyPhen prediction has p-value 0.8788. The HMMvar prediction has a high correlation with the SIFT SNP prediction, but the HMMvar and SIFT SNP predictions both have a weak correlation with the PolyPhen prediction, based on this dataset.

**Table 3:** Dataset from ENSEMBL

		**SIFT**		
		**Deleterious**	**Tolerated**	**Total**
Polyphen	Probably damaging	91	87	178
	Benign + Possibly damaging/PolyPhen	107	108	215
	Total	198	195	393

### Validation on individual proteins TP53

**Figure 5:**
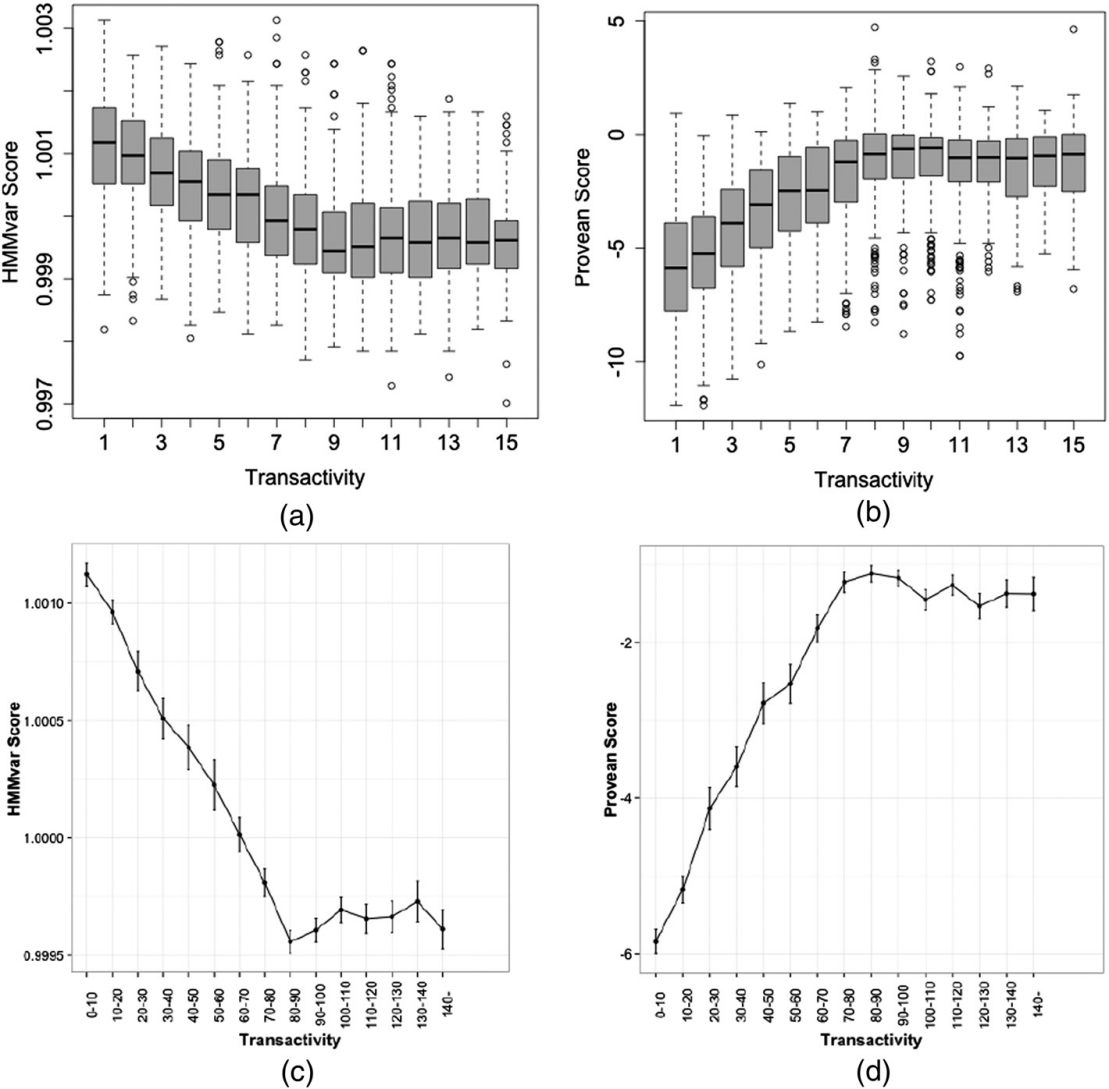
**HMMvar and Provean score distributions and mean/error bars of TP53 mutations binned into 15 classes in terms of transactivity level. (a)** HMMvar score distribution of the 15 classes (x-axis represents the 15 classes based on the median of transactivity levels). **(b)** Provean score distribution of the 15 classes. **(c)** Mean along with error bar of HMMvar scores in each class. **(d)** Mean along with error bar of Provean scores in each class.

**Figure 6:**
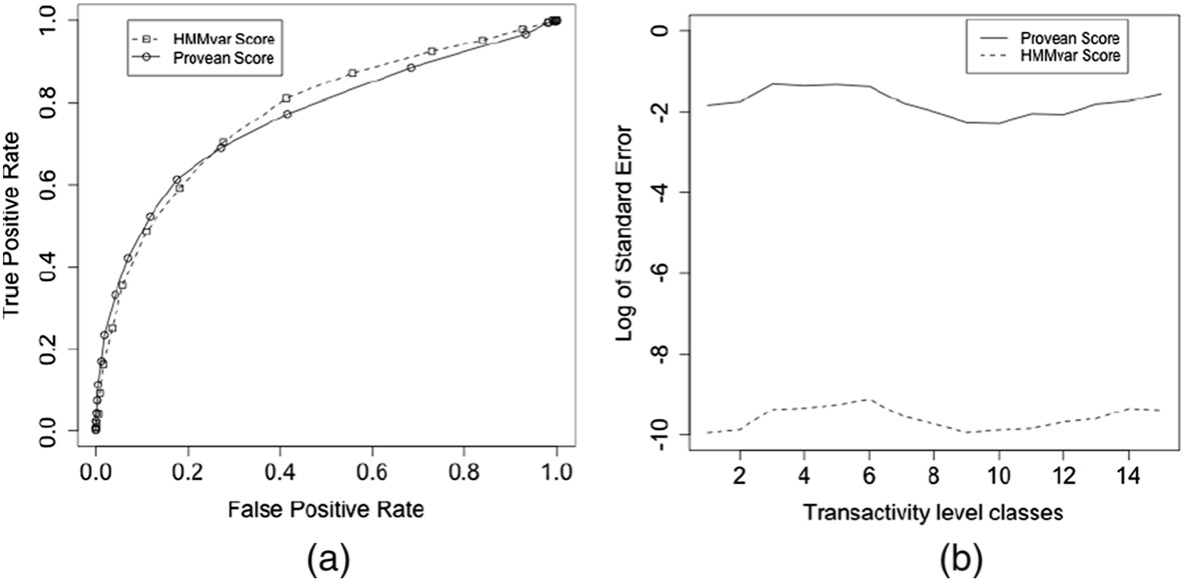
**ROC curve and standard error of the HMMvar score and the Provean score. (a)** ROC curve of the Provean score and the HMMvar score to distinguish "nonfunctional" and "partly functional" classes from "functional" and "supertrans" classes. **(b)** Standard error of the mean of Provean and HMMvar scores in the 15 transactivity level classes.

**Figure 7:**
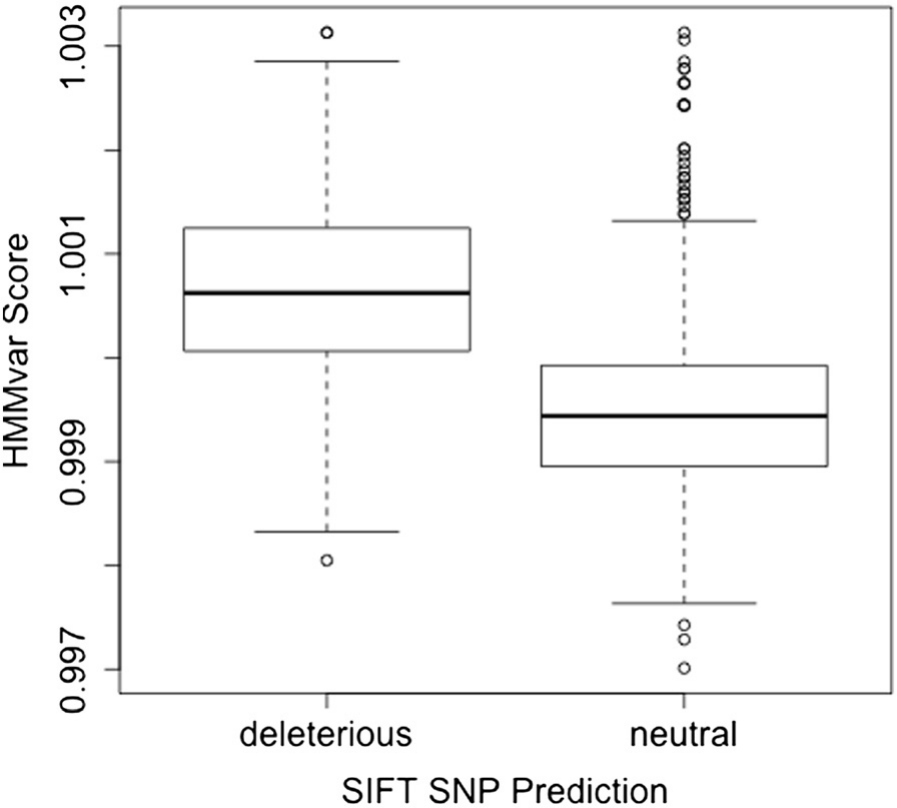
The HMMvar score of TP53 variants grouped by SIFT SNP prediction.

This section addresses whether the HMMvar score can reflect the degree of mutation effects on two extensively studied disease related proteins, TP53 and CFTR. TP53 (known as tumor protein 53) acts as a tumor suppressor, and regulates cell division by keeping cells from growing and dividing too fast or in an uncontrolled way. Single nucleotide variations that cause amino acid changes were divided into 15 functional classes in terms of the median transactivation level of eight different promoters as measured by Kato et al. [25]. For each mutant, the median of the eight promoter-specific activities (expressed as a percent of the wild type protein) is calculated and mutations are classified as "nonfunctional" if the median is < =20, "partially functional" if the median is >20 and < =75, "functional" if the median is >75 and < =140, and "supertrans" if the median is >140. The SNPs are separated into 15 classes in terms of the median values with a increments of 10. The results are also compared with those from another prediction method called Provean [22]. Provean is a recently proposed evolutionary conservation based indel and SNP effects prediction method, which collects a set of homologous sequences to the gene or protein of interest, and then clusters them into different supporting sets to calculate the Provean score based on the delta alignment score. Figure 5(a) and 5(b) show the HMMvar scores and Provean scores vs. the transactivity level, respectively. With respect to the transactivity level, the HMMvar score shows a negative relationship, and the Provean score has a positive relationship, especially in the nonfunctional and partially functional regions. Figure 5(c) and 5(d) show the average scores and error bars for each functional class for the similarity trending HMMvar and Provean scores, respectively. The HMMvar score shows a strong linear relationship with the Provean score (Pearson correlation coefficient *r* = –0.733). The HMMvar score has a slightly lower correlation with the transactivity level (*r* = – 0.523) than the Provean score (*r* = –0.552) but a slightly higher correlation than the SIFT SNP score (*r* = –0.493). Figure 6(a) shows the receiver operating characteristic (ROC) curve for the comparison between HMMvar and Provean in distinguishing "nonfunctional" and "partly functional" classes from "functional" and "supertrans" classes. HMMvar obtained higher AUC (area under the curve) than Provean. To better distinguish between different functional classes, it is highly desirable that a prediction metric exhibits small variance for mutations within the same functional class. Hence consider the variance of HMMvar and Provean scores within each functional class. The standard error of the mean for each functional class is $\text{SE}=\frac{S}{\sqrt{n}}$, where *S* is the standard deviation of the scores for a functional class and *n* is the size of the class. The HMMvar score has much less variance for each functional class as shown by the whisker plots in Figure 5(a,b) and in Figure 6(b), indicating that the HMMvar prediction is more stable than the Provean prediction. There are also SIFT SNP predictions for TP53 variants available in the dataset; comparing the HMMvar score with the SIFT SNP prediction shows that the medians of the HMMvar scores in the two SIFT SNP predicted groups are significantly different (Figure 7).

## Methods

### Dataset description

Insertion and deletion variant data, limited to coding regions, was downloaded from dbSNP Build 137 ( http://www.ncbi.nlm.nih.gov/projects/SNP/) and grouped into two categories, indels with records of disease association in the Locus-specific Mutation Database (LSDB) [24] and those without LSDB records. There are 2631 indels with LSDB annotation and 1458 indels without such records (Table 1). The first disease associated indel group is assumed to be more deleterious than the second one. 393 coding SNPs, for which there are either SIFT SNP or PolyPhen prediction records in Ensembl (Table 3), were used for comparison with the current HMMvar scoring method. For the human tumor suppressor protein TP53, a set of 2,565 SNP mutants and corresponding biological activity levels were obtained from the database IARC TP53 [26]. The mutants associated with TP53 were partitioned into four classes: nonfunctional, partially functional, functional (wildtype), and supertrans (higher activity than wildtype) [25]. Transactivity level was measured by eight promoter-specific activity levels and the classification was made in terms of the median of these eight levels. The dataset CFTR was obtained from the Human Gene Mutation Database (HGMD Professional 2012.3); only SNP mutants were included. The CFTR gene mutants have typical phenotypes, such as cystic fibrosis (CF), congenital absence of vas deferens (CAVD), pancreatitis, etc. This work used only the two largest groups CF (732 single point mutants) and CAVD (98 single point mutants) to test the profile HMM prediction method.

### HMMvar prediction

According to the theory of natural selection, different regions of a functional sequence are subject to different selective pressures. Multiple sequence alignment reveals this by residual conservation in certain positions. Some positions are more conserved than others, and some regions are more tolerant to insertion and deletion variants than others. Mutants occurring at highly conserved residuals are more likely to be deleterious, whereas mutants occurring at lowly conserved residuals are more likely to be neutral or less deleterious. A profile HMM is a nondeterministic finite state machine consisting of a series of states, each of which corresponds roughly to a position (column) in the multiple sequence alignment from which the HMM was built [23]. Scoring (computing the probability of generation by a given Markov process) a wild type sequence or mutated sequence with the profile HMM gives one an idea how far the given sequence is away from the original population. A profile HMM captures the characteristics of a multiple sequence alignment, from which quantitative conservation information (a probability) is obtained. Thus, a high score of the probability of generation from the profile HMM for the wild type sequence and a low HMMvar score for the mutant sequence probably mean that the mutation has deleterious effect.

The five-step prediction pipeline (Figure 1) receives a set of indels (or other types of variants) as input. The first step identifies all unique proteins associated with these indels as wild type sequences (seeds). Since there may be multiple indels associated with one protein and multiple proteins may be involved with one indel, it is more computationally efficient to first identify all the proteins involved. The mutant sequences for a given wild type sequence are obtained by inserting the indels into the wild type sequence. The second step, using the identified proteins as seeds, invokes PSIblast [27] on the nonredundant protein sequence (nr) database to find a set of homologous sequences for each seed protein. The e-value and iteration limits were 0.01 and five, respectively. Only homologous sequences with an identity percentage higher than 90% are used in the next step. The third step invokes ClustalW2 [28] with the BLOSUM62 matrix and the word size three for multiple sequence alignment for each homologous sequences set. The next step builds profile hidden Markov models with HMMER3 [29] using the multiple sequence alignments as training data (one HMM per seed protein). All mutant type sequences derived from the same seed sequence will use the same HMM for functional effect prediction. The last step uses all the constructed HMMs for functional predictions. Precisely, given an input indel (mutant type) corresponding to seed protein *i* (wild type), the *i*th profile HMM is used to compute the HMMvar score *S*, as defined below.

The bit score from HMMER3 measures the similarity of a query sequence with the set of homologous sequences used to define the profile HMM. The HMMER3 bit score is a base 2 logarithm of ratio of probabilities (homology hypothesis over the null hypothesis),

$$B=\text{log}\frac{P\left(O_{1}O_{2}\cdots O_{n}|\text{NMM}\right)}{P\left(O_{1}O_{2}\cdots |\text{NULL}\right)},$$

where *O*_1_*O*_2_…*O*_*n*_ is the observed protein sequence and "HMM" is the trained profile HMM. "NULL" is the "null model", which is a one-state HMM configured to generate "random" sequences of the same length as the target sequence, with each residue drawn from a background frequency distribution. In HMMER3, for proteins, the frequencies of the 20 amino acids are set to the amino acid composition of SWISS-PROT 34 [30]. Since this logarithm score *B* has no direct statistical interpretation, the constituent probabilities are extracted and used to define the HMMvar score as the odds ratio

$$S=\frac{P_{w}/\left(1-P_{w}\right)}{P_{m}/\left(1-P_{m}\right)},$$

where *P*_*w*_ (*P*_*m*_) is the probability that the wild type (mutated type) protein sequence could have been generated by the profile HMM trained on a seed protein homologous sequence set. Usually, this probability is calculated by the Viterbi algorithm. Here, this probability is derived from the bit score obtained from the HMMER3 package. Given a protein sequence, the probability that it was generated under the null model is

$$P_{\text{null}}=\text{exp}\left(l^{*}\text{log}P_{1}+\log\left(1-P_{1}\right)\right),$$

where *l* is the length of the sequence and *P*_1_ is set to 350/351 in the architecture of plan 7 null model [31]. From the null model and bit score equation, the probability *P*_*w*_ or *P*_*m*_ can be derived as *P* = *P*_null_ *e^*B*^ given a wild type sequence or mutated type sequence.

Each wild type sequence (or seed protein) corresponds to one HMM model. Theoretically, the wild type sequence bit score could be less than or equal to zero, however, it makes no sense to compare the mutant type sequences with this wild type sequence, because the wild type itself does not match with the HMM model. Consequently, we consider only the HMMs whose wild type sequence bit scores are greater than zero and compute the odds ratio for mutant type sequences that derive from these wild type sequences. The odds ratio is expected to be greater than 1, indicating the wild type sequence is more likely to occur in the HMM presented family. However, in practice, this is not always the case, which indicates that the mutant type sequence better fits the homology set profile. This situation may result from the nucleotide level mutation causing the amino acid level changes to be more compatible [30] with the homologous sequences than the wild type protein.

If the HMMvar score *S* is less than a threshold *t*, the indel is considered as neutral, otherwise deleterious. Fisher’s exact test was used to choose the threshold, using SIFT indel prediction as the reference method, by minimizing the exact test p-value, giving the optimal threshold *t* = 2.0 for the data sets used.

Instead of the odds ratio *S*, one could use the HMMER3 bit scores directly in the difference

$$D=B_{w}-B_{m},$$

which is the base 2 logarithm of the relative risk (probability of generating the wild type sequence over the probability of generating the mutant type sequence). This was done for the TP53 and CFTR datasets, but the prediction results using *D* were not better than for *S*, and hence are not reported here.

### Parameter selection

The selection of homologous sequences is key to building a high quality profile HMM. The nonredundant protein sequence (nr) database was used with PSIblast [27] to collect homologous sequences for each seed protein, using e-value 0.01, and iteration limit five. All sequences above 90% identity were selected as homologous sequences for a certain seed protein. Attempts to improve diversity in the homologous sequence set by including the sequences below 10% identity or using instead all sequences from 60% identity to 95% identity did not produce better HMMvar score distributions. SIFT SNP prediction is used as a reference to determine HMMvar score thresholds of 2.

## Discussion

Most existing methods for variant effect prediction are based on evolutionary conservation theory, which predicts that highly conserved sites experience strong purifying selection and mutations in these sites are most likely to be deleterious to protein function. However, these methods take each site independent of other sites and do not consider the impact of surrounding sites. Moreover, most of these methods are designed only for SNP variants. In contrast, a profile HMM serves as a representation of a set of homologous sequences, relating all sites through a Markov process. Consequently, the present method HMMvar can provide functional predictions for the effects of all types of sequence variations besides SNPs, and can predict the effect of multiple variants simultaneously. The latter is especially useful as when multiple variants occur in a protein, each one of them may have deleterious effects on protein function, but the combination of them may have less harmful effect due to the possibility of compensatory effect. Profile HMMs, used as proposed, have the capability to predict the total effect of multiple mutations along the gene given a specific haplotype.

### Factors affecting the prediction of indel effect

**Figure 8:**
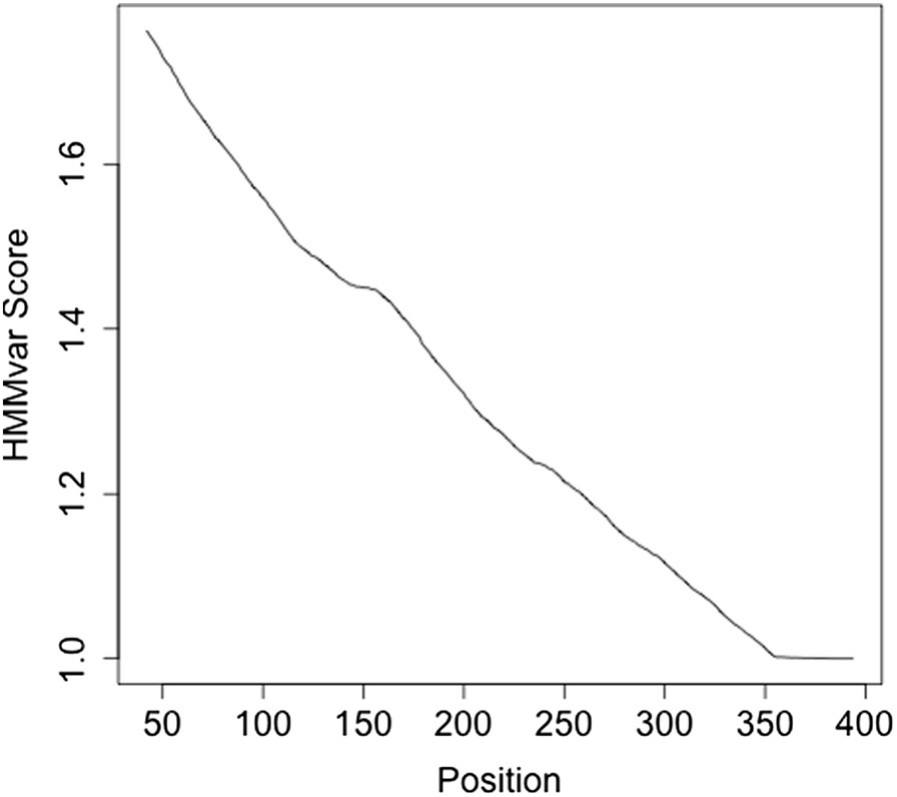
The relationship between the HMMvar score and the position of an artificially introduced variant.

The experiments show several factors that affect the prediction score, such as the location of indels in the protein (Figure 8), and different types of indels (nonsense, missense, or frameshift, Figure 3). It is expected that frameshift indels close to the 5’ end of the sequence are more likely to have deleterious effect than indels occurring close to the 3’ end of the sequence as the former may affect a larger number of amino acids. (Extensive simulation of indels or SNPs introduced at different positions along proteins and subsequent HMMvar predictions confirm this expectation, for brevity, results are not shown here. Figure 8 displays the relationship between HMMvar score and the position of an artificially introduced stop codon to a random protein). Nonsense variants introduce a stop codon at the mutation resulting in the termination of mRNA translation, which brings a greatly deleterious effect if occurring close to the 5’ end of the sequence. A missense mutation may change some amino acids locally, thus may have a relatively smaller effect compared to frameshift or nonsense variants.

It is expected that the quality of multiple sequence alignment is another factor that can potentially affect the prediction of indel effect. Comparing the HMMvar scores based on different multiple sequence alignment programs, ClustalW [28] and MUSCLE [32], for the TP53 transitivity level dataset, showed that HMMvar scores based on the MUSCLE sequence alignment decreases more smoothly and shows lower variance within the same functional classes than scores based on the ClustalW sequence alignment. This suggests that having high quality sequence alignment is important for accurate indel effect prediction.

## Conclusion

With the dramatic increase of the number of genetic variations discovered in human and other species’ populations, much effort is required in order to fully understand their effect on species. This paper proposed a quantitative prediction method, HMMvar, to predict the effect of genetic variation, both indels and SNPs, using hidden Markov models. Results show that HMMvar can achieve good performance in identifying deleterious or neutral variants for different datasets, and can predict the protein functional effects of both single and multiple mutations.

## Competing interests

The authors declare that they have no competing interests.

## Authors’ contribution

ML, LTW, and LZ wrote the paper. ML performed the computational experiments. LTW proposed the HMMvar *S* and *D* scores. LZ proposed the use of HMMs for variant effect prediction. All authors read and approved the final manuscript.
